# Combining Wake-Up-Back-to-Bed with Cognitive Induction Techniques: Does Earlier Sleep Interruption Reduce Lucid Dream Induction Rate?

**DOI:** 10.3390/clockssleep4020021

**Published:** 2022-04-20

**Authors:** Daniel Erlacher, Vitus Furrer, Matthias Ineichen, John Braillard, Daniel Schmid

**Affiliations:** 1 Institute of Sport Science, University of Bern, 3012 Bern, Switzerland; matthiasineichen@hotmail.com (M.I.); john.braillard@students.unibe.ch (J.B.); 2 PHBern, University of Teacher Education, 3012 Bern, Switzerland; vitus.furrer@phbern.ch; 3 Institute of Psychology, University of Bern, 3012 Bern, Switzerland; daniel.schmid2@unibe.ch

**Keywords:** lucid dream induction, wake-up-back-to-bed, MILD, reality testing, sleep laboratory, morning sleep

## Abstract

Lucid dreaming offers the chance to investigate dreams from within a dream and by real-time dialogue between experimenters and dreamers during REM sleep. This state of consciousness opens a new experimental venue for dream research. However, laboratory study in this field is limited due to the rarity of lucid dreamers. In a previous study, we were able to induce in 50% of the participants a lucid dream in a single sleep laboratory night by combining a wake-up-back-to-bed (WBTB) sleep routine and a mnemonic method (Mnemonic Induction of Lucid Dreams, MILD). In three experiments, we tried to replicate our earlier findings while we adapted our procedure in shortening (Exp1–3: 4.5 vs. 6 h of uninterrupted sleep in the first half of the night), simplifying (Exp2: time-based wakening vs. REM wakening in the second half of the night), and applying another induction technique (Exp3: reality testing vs. MILD). In the three conditions, four out of 15 (26%), zero out of 20 (0%), and three out of 15 (20%) participants reported a lucid dream. Compared to the original study, the earlier sleep interruption seems to reduce the lucid dream induction rate. Furthermore, without REM awakenings in the morning, lucid dream induction failed, whereas reality testing showed a lower success rate compared to MILD. Further systematic sleep laboratory studies are needed to develop reliable techniques for lucid dream research.

## 1. Introduction

Lucid dreaming is defined whereby a dreaming person is fully aware of his or her current dream state and this state of consciousness often leads to increased volitional control over the ongoing dream and full access to memory [1]. Proficient lucid dreamers can perform pre-arranged eye movements in their dreams, which can be validated in a sleep laboratory setting [2,3]. This signalization by eye movement during lucid dreaming allows the study of psychophysiological correlations between dreamed and executed actions in sleep laboratory studies [4,5]. Furthermore, by real-time dialogue between experimenters and dreamers during REM sleep [6,7], this state of consciousness opens a new experimental venue for dream research in general [8,9]. A major challenge in lucid dream research remains the reliable induction of lucid dreams [10]. In the general population, the percentage of individuals experiencing lucid dreams on regular basis (once a month or more frequently) is 20%, yet only 1% have lucid dreams several times a week [11,12]. However, lucid dreaming is a learnable ability [10,13] and several techniques have been described to induce lucid dreams, which can be roughly distinguished in cognitive techniques (e.g., reality testing or reflection technique) or approaches using external stimulation [10,14]. Even though there is evidence for the effectiveness of cognitive techniques, the success rate of most field studies is relatively small [14]. The idea behind the external stimulation is to present a stimulus to the sleeping person, e.g., using auditory stimulation [15,16], visual or tactile stimulation [17], or olfactory-cued reactivation [18], during REM sleep. A combination of cognitive methods and external stimulation seems to be promising to increase lucidity. In a sleep lab research, Carr et al. [19] coupled visual and audio stimulation during REM sleep with reality checking and mindfulness during a morning nap. In the experimental group, 50% of the participants had a lucid dream verified by volitional eye signaling, whereas in the control group, only 17% of the participants reported a lucid dream. However, Schmid and Erlacher [15] found in a sleep laboratory study that reality testing combined with auditory stimulation results in having 14% of the participants experiencing a lucid dream, which is significantly below the success rate of Carr et al.’s induction techniques. It seems that small methodological changes between studies with similar techniques result in dramatic differences in the lucid dream success rate. 

Another promising approach seems to be the combination of a wake-up-back-to-bed sleep protocol (WBTB) and dream work (e.g., Mnemonic Induction of Lucid Dreams, MILD). In a previous study, we conducted a sleep laboratory experiment with four distinct experimental conditions whereas the experimental design was as follows [20]: After 6 h of sleep, participants were woken from a subsequent REM phase and remained awake for 30 or 60 min, during which they were instructed to do MILD or a control task (e.g., reading). They then went back to bed for a morning nap. In the two conditions with 60 min MILD intervention 53% of the participants experienced a lucid dream and 27% produced polysomnography-verified eye signals. In contrast, in the control condition, only one (9%) participant reported lucid dreams and no eye movements. No lucid dreams were observed in the Wii condition [21]. Our findings were replicated by another sleep laboratory study [22]. 

Because the aforementioned experimental procedure was very effective in inducing lucid dreams, it also lasted for up to 11 h from the beginning of the night (“lights out”) until the final awakening. Thus, we reduced the time spent in bed for the first half of the night. Instead of 6 h (4 REM periods), we reduced the time to about 4.5 h (3 REM periods) of uninterrupted sleep. This change was made for all three experiments. Furthermore, the protocol for the REM awakenings in our original study was rather complex for the second half of the nights with three different rules for REM awakenings [20]. Therefore, we simplified the procedure in the second half of the night to an awakening independent of sleep stage (e.g., REM sleep) just on a time base (e.g., 8:00 am). Finally, we were interested in whether other induction techniques would show similar results in the combination with WBTB. Therefore, in experiment 3, we conducted a reality testing intervention during the wake period instead of MILD. For all experimental conditions, we expected lucid dream induction rates of about 50%.

## 2. Results

### 2.1. Sleep Data 

Table 1 depicts the sleep data for the second half of the night. Of the total 50 experimental sleep nights in this study, 48 (96%) resulted in usable polysomnography (PSG) recording. In experiments 1 and 2, the PSG recording was unspecifiable for one participant each. All the remaining 48 participants were able to fall asleep after WBTB, with a sleep latency of 12.0 ± 7.4 min. Each participant showed at least one REM period, up to a maximum of five after WBTB, with a REM latency of 57.9 ± 20.3 min after lights out.

### 2.2. Dream Reports

In total, 73 dream reports (Exp1 = 25, Exp2 = 24, Exp3 = 24) were collected for all experimental nights. For the first half of the night, 39 dreams (Exp1 = 11, Exp2 = 18, Exp3 = 10) were reported after REM awakenings with a total dream recall rate of 78% (Exp1 = 73%, Exp2 = 90%, Exp3 = 67%). For the second half of the night, 28 dreams (Exp1 = 14, Exp2 = 6, Exp3 = 14) were reported in the morning either after REM awakenings (Exp1 and 2) or time-based awakenings (Exp2); whereas in Experiment 1, one participant, in Experiment 2, two participants, and in Experiment 3, three participants reported two dreams. The overall dream recall rate for the number of participants is 56% (Exp1 = 87%, Exp2 = 20%, Exp3 = 73%). The dream reports were rated for lucidity by two independent raters. The average length of a dream report consisted of 122.9 ± 90.5 words. The interrater reliability showed almost perfect agreement in experiments 1 and 3 and substantial agreement in experiment 2, with an average weighted kappa of κ1 = 0.833, κ2 = 0.623, and κ3 = 0.925.

### 2.3. Induction of Lucid Dreams

Overall, seven participants (14%) reported lucid dreaming after WBTB. One participant (2%) was not sure whether the dream was lucid and if the LRLR-eye signal had been given. Further, 42 participants (84%) did not report a lucid dream. The judges rated 62 dream reports without evidence of lucid dreaming (1–2 on Stewart & Koulack lucidity scale [23]), four with indications of lucid dreaming (3 on Stewart & Koulack lucidity scale [23]), and seven with evidence of lucid dreaming (4–6 on Stewart & Koulack lucidity scale [23]). The dream report of the person who was unsure about the lucidity was rated by the judges as with indications of lucid dreaming. All seven dreams reported by the participants as lucid were also rated as lucid by the judges. Out of these seven participants, five had an unambiguously left-right-left-right (LRLR) eye signal verified by PSG recording. The remaining two participants did not show any prearranged eye movement after WBTB, as well as all the other participants. The numbers of lucid dreams recorded across the three experiments are listed, according to the criteria “strict” and “loose”, in Table 2.

In experiment 1 (4.5 h + 1 h MILD + REM awakening) four out of 15 participants (26%) reported having a lucid dream. The external judges rated three as lucid dreams and one as a dream with indications of lucidity. Two of these four PSG recordings showed clear prearranged eye movement (13%), whereas the other two showed only an ambiguous (/questionable) LRLR-eye signal (13%). In experiment 2 (4.5 h + 1 h MILD + time-based awakening) none out of 20 participants reported having a lucid dream. The external judges rated none of the dream reports as lucid nor with an indication of lucidity. Hence, no eye signal could be verified. And finally in experiment 3 (4.5 h + 1 h RT + REM awakening) three out of 15 participants (20%) reported having a lucid dream. The external judges rated all three as lucid dreams. On each of these three PSG recordings, an unambiguous LRLR-eye signal could be verified (20%).

## 3. Discussion

The findings of the present study show that we were not able to replicate our previous results with respect to the lucid dream induction rate by applying a combination of WBTB and MILD [20]. The low induction rate in this study could be explained by the fact that several methodological changes have been intentionally made compared to our previous work with higher induction rates, and therefore those changes should be discussed in detail.

Firstly, the sleep duration in the first half of the night was shortened from the previous 6 h to 4.5 h of sleep. The reduction of sleep duration until the WBTB intervention could lead to circadian and homeostatic differences that we did not measure in our study. In a similar sleep laboratory study with additional odor stimulation by our research group [18], we also applied a sleep interruption after 4.5 h in combination with MILD, which led to a reduction in lucid dream success rates (12.5%). Because of the shift of the intervention of about 90 min, the following REM sleep might be less prominent in the second half of the night. However, in this study, the second half of the night showed short REM latencies and long REM durations (see Table 1). Therefore, this explanation seems rather unlikely. However, in further studies, the different time sets should be experimentally tested in a within subject design to clarify the effect.

Furthermore, due to the earlier sleep interruption, it might be the case that the participants still show high sleep pressure in the second half of the night. All participants in this study revealed high sleep efficiency (>90%) for the second half of the night, whereas in our previous study [20] the sleep efficiency was between 66% and 83%. One possible explanation for this might be that lighter sleep has a beneficial effect on lucid dream induction [24] and therefore could explain the lower induction rate in the this study. However, the relationship between lighter sleep and lucidity seems to be rather optimally linear, e.g., in cases where participants cannot sleep at all or do not show REM sleep after WBTB, when the procedure is apparently negatively associated to lucid dream induction. The underlying processes that are responsible for lighter sleep (e.g., hormonal factors) and why such processes should promote lucidity are not well understood and further research is needed.

Secondly, the induction session had been modified in the third experiment from a MILD procedure [25] to reality testing technique [26]. Although both techniques differ in their instructions, they both depend on prospective memory. Still, the differences in the induction session might harm the lucid dream induction rate. This is underlined by the aforementioned study by our research group in which we combined WBTB, reality testing, and odor stimulation leading to three out of 15 participants (20%) experiencing a lucid dream in a single sleep laboratory night. Nevertheless, systematic research on the content and effectiveness of different cognitive procedures, e.g., MILD or reality testing, is scarce [27].

Thirdly, the REM awakening procedure for the second half of the night was simplified for experiment 2 which showed no lucid dreams in a cohort of 20 participants. This null effect is rather surprising because the rest of the procedure was kept similar to all other experiments. One effect of this simplification was that the dream recall rate for the morning awakenings dropped to 20%. One might speculate that this experiment also provoked some lucid dreams. However, the participants were unable to recall the content. This assumption is supported by the general idea that a low dream recall rate results in low lucid dream rates [28]. However, in sleep laboratory studies with REM awakenings, the participants will usually have the impression that the recalled dream is from the REM phase they were awakened from, and therefore it seems likely that some lucid dreams might have been lost because of specific REM awakenings. Therefore, in future studies, REM awakenings should be applied systematically in the second half of the night. However, clear criteria have to be developed when the REM awakening should be performed because it might be the case that the awakening might interfere with the development of lucidity in an ongoing dream.

A final methodological limitation that needs to be addressed in future sleep lab research is the proper validation of lucid dream by LRLR eye signals, because during a night recording participants might show hundreds of eye movements during (REM) sleep. Therefore, a high probability exists to find by chance a LRLR [15]. One methodological approach would be to compare each LRLR sequence against the probability to find it by chance. Since no signal verified lucid dream was induced in this study, this empirical evaluation has not been carried out in this study but is recommend for future lab research in the lucid dream induction field [16].

In dream research, neural correlates are often discussed in association with dream recall rate (e.g., [29]) or with factors of lucidity [4]. However, it might be possible that circadian factors also play an important role and interfere with dream recall rate and lucidity. Therefore, in further studies, such circadian factors as the time of night, chronotype, light intensity during the intervention, melatonin production, or menstrual cycle in women should be controlled experimentally.

To summarize, the present study combined the so-called wake-up-back-to-bed sleep protocol (WBTB) after 4.5 h and MILD/reality testing in three experiments to induce lucid dreams. From a total of 50 participants in three different experiments, the procedure induced in seven participants a lucid dream, whereas five of those lucid dreams were verified by an LRLR eye signal. The success rate of a combination therefore could not replicate the high success rate of other similar induction techniques in earlier studies. Future studies should focus on the methodological factors raised and their influence on lucid dream induction.

## 4. Materials and Methods

### 4.1. Participants

In the first experiment, 15 students (9 male, 6 female, 25.1 ± 2.4 years), in the second experiment, 20 students (10 male, 10 female; 24.7 ± 1.5 years), and in the third experiment, 15 students (12 male, 3 female; 25.5 ± 2.00 years) participated. The age of the total 50 participants ranged from 23 to 31 years (see Table 3). All participants were sport students from the University of Bern. Participation was voluntary and unpaid, and students received course credits in return, which could also be obtained with other courses. Thus, the participants were self-selected by their interest in the field of sleep, dreams, and sports. For the female participants, menstrual cycle was not checked. At the time of data collection for experiments 1 and 2, ethical review and approval was not required for the study on human participants in accordance with the local legislation and institutional requirements. For the third experiment, the faculty of Human Sciences Ethics Commission of the University Bern approved the protocol (Nr. 2016-06-00002). All participants provided written, informed consent before the beginning of the study and the experiment was conducted in accordance with the Declaration of Helsinki.

### 4.2. Dream Recall and Lucid Dream Recall Frequency

For obtaining data about dream and lucid dream recall frequency, the participants filled out the Mannheim Dream questionnaire (MADRE [30]). The dream recall frequency was estimated on a 7-point rating scale developed by Schredl [31], ranging from 0 = never to 6 = almost every morning. Its retest reliability is high (r = 0.85). By recoding the scale to class means, units of dreams per week were attained (0 = 0, 1 = 0.125, 2 = 0.25, 3 = 0.625, 4 = 1.0, 5 = 3.5, 6 = 6.5). Likewise, lucid dream frequency was estimated on an 8-point scale ranging from 0 = never to 7 = several times a week. This scale was also recoded to class means, to obtain the units of lucid dreams per month (0 = 0, 1 = 0.042, 2 = 0.083, 3 = 0.25, 4 = 1.0, 5 = 2.5, 6 = 4.0, 7 = 18). According to Snyder and Gackenbach (1988), a clear understanding of the definition of lucid dreaming is crucial for the measurement of subjective lucidity. Therefore, a definition is provided: “In lucid dreams, one has awareness that one is dreaming during the dream. Thus, it is possible to wake up deliberately, or to influence the action of the dream actively, or to observe the course of the dream passively” (Erlacher, 2010, p. 20). Participants’ understanding is tested by them explaining the definition before sleep.

### 4.3. Polysomnography

Data about the sleep composition was gathered with continuous polysomnography (PSG). PSG recording included electroencephalogram (EEG: F3, F4, C3, C4, O1, O2), electrooculogram (EOG), submental electromyogram (EMG), including electrocardiogram (ECG). The EEG electrodes were placed according to the Ten-Twenty system (Jasper, 1958). A standard sleep recording device (XLTEK Trex Longtime EEG recorder; nbn Medizin Elektronik GmbH, Soltau, Germany) recorded sleep data all night. The sleep recordings were manually scored in sleep stages according to the AASM criteria (Iber, Ancoli-Israel, Chesson, & Quan, 2007). The following sleep parameters were of interest: total bed time (min), total sleep time (min), sleep efficiency (%), sleep latency (min), REM latency (min), REM period count, REM period range, REM total time (min), REM% SPT, Wake% SPT, Stage1% SPT, Stage2% SPT, Stage3% SPT.

### 4.4. Mnemonic Induction of Lucid Dreams (MILD)

The MILD technique uses the ability of the prospect memory, which allows to set an intention or reaction based on events to be experienced in the future. This technique is the one most often tested empirically and first used in a sleep laboratory study [14]. MILD is most useful after spontaneous awakening with dream recall, because this allows to set an intention to become lucid the next time bizarre or unrealistic events occur during the dream [20]. MILD was introduced to the participants for experiments 1 and 2, and during the intervention it was practiced. It consisted of three steps: (1) writing the dream report, (2) finding dream signs, and (3) practicing MILD. While lying in bed after the intervention, the participants continued with step three, visualizing themselves becoming lucid the next time they see a dream sign.

### 4.5. Reality Testing

Reality testing (RT) was first introduced by Tholey in 1982 [26], where he introduced a 10-step guide to induce lucid dreaming using reality testing. The reality test is a technique in which one repeatedly asks oneself concretely and seriously in an awake state whether one is awake or in a dream state. This critical attitude towards reality can be transferred into the dream world if it is carried out sufficiently. Thus, one can become lucid by the critical questioning of incongruent occurrences in the dream [26]. Reality testing was introduced to the participants for experiment 3, and during the intervention period they followed a three-step practice: (1) reading information about the induction of lucid dreams via critical reflection, (2) performing reality tests, and (3) repeating and internalizing reality tests.

### 4.6. Procedure

Participants spent a single night in the sleep laboratory in Bern, in a dark and quiet room. All participants received full information regarding the procedure and aim of the studies. Written informed consent was obtained by all participants, right after they arrived in the sleep laboratory around 9:00 pm. Immediately afterwards, they were familiarized with the premises. In a next step, the electrodes for PSG were attached, and the participants prepared themselves for the night. After assuring correct sleep recording, the left-right-left-right eye movements (LRLR) were explained to the participants, followed by testing whether a clear LRLR eye signal could be successfully identified on the sleep recording, which is used to signal a possible lucid dream (LaBerge et al., 1981). Participants were informed that they would now have at least 4.5 h of uninterrupted sleep. The subsequent night procedure can be divided into four parts:

First half of the night: In experiments 1 and 2, the participants were only awakened after 4.5 h of sleep and a stable REM interval of at least 10–15 min If in experiments 1 and 2 the participants did not have any stable REM of 10–15 min after 4.5 h of sleep, then they were awakened when the REM period was at least 7 min long. The limit was set after 5.5 h of falling asleep, and any REM period, no matter how long, resulted in the awakening of the participant. The last rule for awakening was that when the experimenter recognized a LRLR eye signal, then the participants were awakened three epochs (each 30 s) later.

Awakening: In experiment 1, the participants were awakened by knocking on the door and calling their name until they responded. In experiments 2 and 3, the participants were called by their name via an intercom system until they responded. They were immediately asked to report any mental content that was in their mind before awakening. To assure that the participants had enough time to remember the mental content, they were given up to 2 min to recollect their dream experience. When the participant reported a dream, he was asked whether he was aware of his dream state and if he executed a LRLR eye signal. The whole conversation was recorded via voice recorder. The lights were turned on and the experimenter went in the room of the participant where the intervention was implemented.

Intervention: In Experiments 1 and 2, the lucid dream induction techniques used were WBTB in combination with MILD. The participants first had to write down their dream report (15 min), which is needed for the implementation of MILD. In a second step, a motivation letter about experiencing a lucid dream in the sleep laboratory was read and filled out (5 min). The third step was to search the dream for dream clues. Therefore, the participants were given an information sheet about dream clues. Then, they had to analyze their created dream reports for such dream clues (20 min). If the participant could not remember the dream and no dream report was made, then dream reports from the dream diary were used, in which the participants had a record of their dreams for at least the three nights before the sleep laboratory night. The last step included giving the participants an information sheet about MILD. To assure that the participants had understood the meaning, they had to explain it to the experimenter. For the remaining time, MILD was practiced lying in bed (20 min), and just before turning the lights off, the LRLR eye signal was practiced again.

In experiment 3, a combination of WBTB and reality testing was used to induce lucid dreaming. First, the participants were given an information sheet about lucid dreaming via reality testing (5 min). Next, the participants received information about three specific types of reality test, of which three versions existed (1.1 Reading, 1.2 Turning, 1.3 Breathing; 2.1 Hand, 2.2 Jump, 2.3 Light switch; 3.1 Time, 3.2 Wall, 3.3 Own experience). Versions 1–3 were equally distributed between participants. The three specific reality tests were solidified by explaining and showing them to the experimenter (25 min). Subsequently, the reality tests were repeated and internalized with two tasks. The first was watching an 11-min film sequence from “waking life” on a laptop. During that time, they had to perform, at least twice, the three reality tests in the waking world. For the remaining time, the three reality tests learned were performed in bed five times with eyes open (reality) and five times with eyes closed (imagined dream world). The intervention in all three experiments lasted around 1 h. The time frames were used for reference only. Interindividual differences for the correct and conscientious completion of the tasks were tolerated.

Second half of the night (back to bed): During the second sleep period of the participants, the experimenters of all three studies were awake. The participants were awakened three periods after the occurrence of a LRLR-eye signal, whereas the awakening followed the same procedure as in the awakening before intervention. If no LRLR-eye signal was observed by the experimenter, then different rules of awakening applied. In experiments 1 and 3 the participant was awakened after a 15-min continuous REM phase, after 3 h of falling asleep. If the REM phases were too short, then after 4 h of falling asleep, a shorter REM period resulted in the awakening of the participant. In experiment 2, the participants were awakened after every continuous REM period of 5 min. In all three studies, the dream reports were recorded with a voice recorder. In experiments 1 and 3, dream reports were also written down on a dream report sheet.

### 4.7. Criterion for Successful Lucid Dream Induction

The lucid dream induction counts as successful if three different types of proof hold true (for reference, see [20]): (1) the participants subjective self-rating of lucidity, (2) the dream report rated by an external judge as either with possible or clear signs of lucidity, (3) the participant reports a LRLR-eye signaling, which can be unambiguously identified on the PSG recording by an external rater. For the “strict” criterion of successful lucid dream induction, all three proofs must hold true. For the “loose” criterion, (1) and (2) were considered as sufficient.

### 4.8. Statistical Analysis

The free software “R” (version 4.0.3) was used to calculate the interrater reliability coefficient kappa to assure coherent objective rating of dream reports. Differences in mean values in the sleep data between the three groups was calculated using ANOVA (with Variances not assumed equal: Welch’s) and post-hoct tests were performed with Games-Howell (unequal variances).

## Figures and Tables

**Table 1:** Sleep data for the second half of the night of all the participants (experiment 1–3).

	Study Condition				
	1 (4.5 h w/REM + 1 h MILD)	2 (4.5 h w/REM + 1 h MILD)	3 (4.5 h w/o REM + 1 h RT)	ANOVA	
	*n* = 14	*n* = 19	*n* = 15	*F*	*p*
Total bed time (min)	184.7 ± 50.6	202.3 ± 35.1	210.6 ± 54.8	0.95	0.399
Total sleep time (min)	174.7 ± 47.0	189.7 ± 33.5	190.2 ± 51.1	0.55	0.586
Sleep efficiency (%)	94.7 ± 2.6 ^a^	93.8 ± 3.2 ^a^	90.3 ± 4.9 ^b^	4.43	0.021 *
Sleep latency (min)	7.8 ± 3.6 ^a^	17.7 ± 13 ^b^	10.7 ± 7.1	5.54	0.009 *
REM latency (min)	59.4 ± 20	58.2 ± 20.9	56.8 ± 21.5	0.05	0.954
REM period count	2.5 ± 1.0	2.8 ± 0.8	2.7 ± 0.7	0.59	0.560
REM period range	1–5	1–4	1–4	-	-
REM total time (min)	45.7 ± 19.3	49.0 ± 15.6	53.3 ± 15.1	0.71	0.500
REM % SPT	23.8 ± 6.6	24.1 ± 6.5	25.8 ± 5.3	0.47	0.630
Wake % SPT	5.3 ± 2.6 ^a^	6.2 ± 3.2 ^a^	9.7 ± 4.9 ^b^	4.29	0.024 *
Stage 1 % SPT	10.0 ± 3.2 ^a^	27.3 ± 10.3 ^b^	9.6 ± 4.6 ^a^	24.86	<0.001 **
Stage 2 % SPT	55.7 ± 9.3 ^a^	37.8 ± 11.0 ^b^	49.9 ± 7.1 ^a^	12.84	<0.001 **
Stage 3 % SPT	5.3 ± 6.8	4.5 ± 6.2	5.0 ± 4.9	0.05	0.953

Note. MILD = Mnemonic Induction of Lucid Dreams; RT = Reality Testing; ^a^ statistically significant different from ^b^; * *p* < 0.05, ** *p* < 0.01;

**Table 2:** Number and type of lucid dreams in the three experiments.

	Study Condition		
	1 (4.5 h w/REM + 1 h MILD)	2 (4.5 h w/REM + 1 h MILD)	3 (4.5 h w/o REM + 1 h RT)
*n* (male/female)	15 (9/6)	20 (10/10)	15 (12/3)
LD (loose) ^a^ (male/female)	4 (2/2)	0 (0/0)	3 (2/1)
LD (strict) ^b^ (male/female)	2 (1/1)	0 (0/0)	3 (2/1)

Note. ^a^ Number of lucid dreams with loose criterion; ^b^ Number of lucid dreams with strict criterion.

**Table 3:** Participants characteristics.

	Study Condition			Test Statistic	*p*
	1 (4.5 h w/REM + 1 h MILD)	2 (4.5 h w/REM + 1 h MILD)	3 (4.5 h w/o REM + 1 h RT)		
N (male/female)	15 (9/6)	20 (10/10)	15 (12/3)	*χ*^2^(2) = 3.31	0.191
Age	25.13 ± 2.39	24.65 ± 1.49	25.47 ± 1.96	*F*(2,46) = 2.85	0.068
DRF ^a^ (dreams/week)	2.51 ± 1.63	2.71 ± 2.05	2.13 ± 1.84	*F*(2,46) = 0.38	0.686
LDRF ^b^ (lucid dreams/month)	0.09 ± 0.24	0.20 ± 0.55	0.24 ± 0.61	*F*(2,46) = 0.34	0.717

Note. ^a^ Dream recall frequency; ^b^ Lucid dream recall frequency.

## Data Availability

The data from this study can be requested by the corresponding author.
